# *Hypoxylon pulicicidum* sp. nov. (Ascomycota, Xylariales), a Pantropical Insecticide-Producing Endophyte

**DOI:** 10.1371/journal.pone.0046687

**Published:** 2012-10-09

**Authors:** Gerald F. Bills, Victor González-Menéndez, Jesús Martín, Gonzalo Platas, Jacques Fournier, Derek Peršoh, Marc Stadler

**Affiliations:** 1 Fundación MEDINA, Centro de Excelencia en Investigación de Medicamentos Innovadores en Andalucía, Parque Tecnológico de Ciencias de la Salud, Armilla, Granada, Spain; 2 Las Muros, Rimont, Ariège, France; 3 Biology, Chemistry and Earth Sciences, Dept. Mycology, University of Bayreuth, Bayreuth, Germany; 4 Helmholtz Centre for Infection Research GmbH, Dept. Microbial Drugs, Braunschweig, Germany; Unidad de Microbiologua -, Universidad Rovia, Spain

## Abstract

**Background:**

Nodulisporic acids (NAs) are indole diterpene fungal metabolites exhibiting potent systemic efficacy against blood-feeding arthropods, e.g., bedbugs, fleas and ticks, via binding to arthropod specific glutamate-gated chloride channels. Intensive medicinal chemistry efforts employing a nodulisporic acid A template have led to the development of N-*tert*-butyl nodulisporamide as a product candidate for a once monthly treatment of fleas and ticks on companion animals. The source of the NAs is a monophyletic lineage of asexual endophytic fungal strains that is widely distributed in the tropics, tentatively identified as a *Nodulisporium* species and hypothesized to be the asexual state of a *Hypoxylon* species.

**Methods and Results:**

Inferences from GenBank sequences indicated that multiple researchers have encountered similar *Nodulisporium* endophytes in tropical plants and in air samples. Ascomata-derived cultures from a wood-inhabiting fungus, from Martinique and closely resembling *Hypoxylon investiens,* belonged to the same monophyletic clade as the NAs-producing endophytes. The hypothesis that the Martinique *Hypoxylon* collections were the sexual state of the NAs-producing endophytes was tested by mass spectrometric analysis of NAs, multi-gene phylogenetic analysis, and phenotypic comparisons of the conidial states. We established that the Martinique *Hypoxylon* strains produced an ample spectrum of NAs and were conspecific with the pantropical *Nodulisporium* endophytes, yet were distinct from *H. investiens*. A new species, *H. pulicicidum*, is proposed to accommodate this widespread organism.

**Conclusions and Significance:**

Knowledge of the life cycle of *H. pulicicidum* will facilitate an understanding of the role of insecticidal compounds produced by the fungus, the significance of its infections in living plants and how it colonizes dead wood. The case of *H. pulicicidum* exemplifies how life cycle studies can consolidate disparate observations of a fungal organism, whether from environmental sequences, vegetative mycelia or field specimens, resulting in holistic species concepts critical to the assessment of the dimensions of fungal diversity.

## Introduction

Arthropod ectoparasites can cause skin diseases and vector infectious pathogens. In dogs and cats, fleas are the most important ectoparasites worldwide, especially the ubiquitous cat flea (*Ctenocephalides felis*). Flea infestation annoys and causes discomfort to animals and humans and can contribute to anemia and allergic dermatitis in dogs and cats. Fleas may vector a number of pathogenic bacteria, e.g., *Rickettsia typhi*, *R. felis*, and *Bartonella henselae*, and can be an intermediate host for the dog and cat tapeworm *Dipylidium caninum*. To alleviate the morbidity and discomfort associated with these infestations, substantial resources have been directed at identifying safe and effective ectoparasite cidal agents.

**Figure 1:**
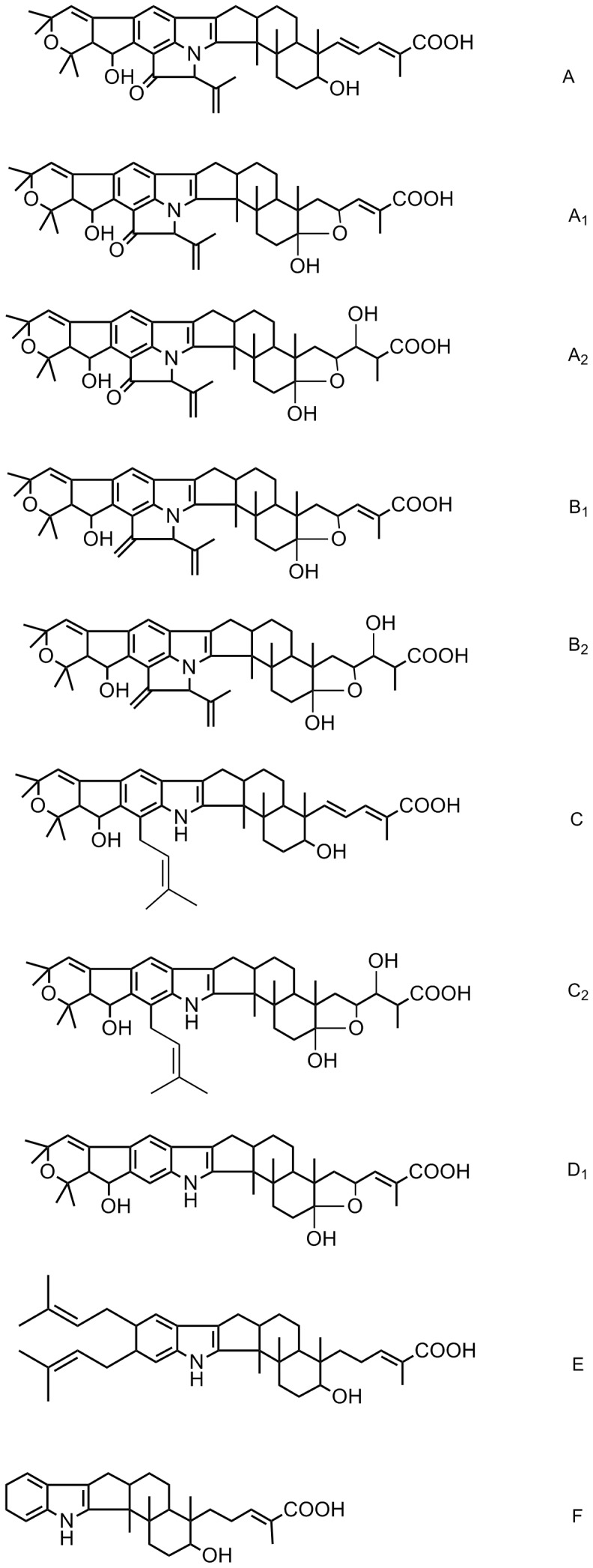
The structures of nodulisporic acids unequivocally detected during this study. See [12] for a detailed description of structures of the nodulisporic acids and their stereochemistry.

Nodulisporic acids (NAs, Fig. 1) are novel indole diterpenes that exhibit potent insecticidal properties and were discovered at Merck Research Laboratories (MRL) in 1992 by screening for activity against the larvae of the blowfly *Lucilia sericata* [1], [2], [3], [4]. NAs not only kill blowflies, but also mosquitoes (*Aedes aegypti*) and fruit flies (*Drosophila melanogaster*). In the blowfly and mosquito assays, nodulisporic acid A (NA A, Fig. 1) was more potent than paraherquamide and abamectin, but less potent than ivermectin [1], 5. Their systemic ectoparasiticide activity was discovered in a bedbug (*Cimex lecularius*) mouse assay [6], [7]. NA A was about 10-fold more potent than ivermectin in a dog flea (*Ctenocephalides canis*) model [5], [8]. As a result, this family of compounds became the focus of a major medicinal chemistry development program directed at long-acting oral cidal agents for control blood-sucking ectoparasites of companion animals [5], [7], [9]. The systemic therapeutic approach has been recently validated by registration in the United States of an oral formulation of spinosad (Comfortis®), a mixture spinosyns A and D, with rapidly acting systemic cidal activity for the treatment of fea infestations of dogs [10].

NAs belong to a common fungal metabolite family characterized by a hybrid molecular architecture consisting of an indole nucleus and cyclic diterpenoid moiety with high structural diversity [11]. Several NAs have been described [2], [12], [13], [14], with the most potent being NA A (Fig. 1). Natural indole diterpenoids exhibit interesting biological properties including tremorgenic and insecticidal activities [15], as well as antibacterial effects against methicillin-resistant *Staphylococcus aureus* [16]. However, despite their strong structural resemblance to tremorgenic indole diterpenes, the NAs biology is unique, exhibiting potent systemic activity against fleas and ticks in cats and dogs, yet are devoid of acute mammalian toxicity [5], [7].

NA A modulates arthropod-specific glutamate-gated chloride ion channels in a similar fashion to ivermectin; however unlike the ivermectins, it does not interact with GABA-gated chloride channel [5], [17], [18]. Disruption of these ion channels silences neuronal activity, paralyzing insects, while having no effect on mammals because homologous targets are absent. Systematic synthetic modification of NA A eventually identified pharmacologically permissive and nonpermissive regions of the natural product [19] leading to semisynthetic derivatives with enhanced cidal flea efficacy based on amide analogs, called nodulisporamides [9]. Modular synthesis produced a library of 355 nodulisporamide derivatives for evaluation, eventually centering on N-*tert*-butyl nodulisporamide as a product development candidate. This derivative has enhanced potency and extended half life, with single oral dose protecting cats and dogs against fleas and ticks for a month or more [7].

NAs have only been known to be produced by a monophyletic lineage of asexual endophytic fungal strains widely distributed in the tropics and tentatively identified as a *Nodulisporium* species [4], [20]. These strains were morphologically similar to the asexual states of fungi in the Hypoxyloideae of the Xylariaceae, especially certain *Hypoxylon* species. More recently this lineage of *Nodulisporium* strains was hypothesized to represent a unique phylogenetic and chemotypical lineage within the Hypoxyloideae [21], [22], [23]. Functional screening focused on a specific insect receptor assay discriminated a few newly isolated strains from among the thousands of fungal isolates over the course several years and recognized a group of phylogenetically cohesive NAs-producing strains. High throughput detection of chemical phenotypes differs radically from taxon recognition based on conventional taxonomic grouping of field and museum specimens, with the recognition process perhaps being more similar to the recognition of an unknown and emerging pathogen causing a plant or animal disease [24]. Furthermore, inferences from GenBank sequences suggested that other researchers have independently encountered tropical *Nodulisporium* endophytes similar to those isolates discovered at MRL, and likewise were unable to link these strains to a known taxonomic entity.

Fungi of the Xylariaceae have almost exclusively been classified and named based on their sexual reproductive states, while cultural phenotypes of the Xylariaceae obtained independently of the sexual state have been considered to lack sufficient information for classification [25], [26], [27]. Although synchronization of molecular markers between ascomata-derived and endophytic strains may overcome some of these perceived limitations [28], [29]. Therefore, two alternatives might explain the failure to name the NAs-producing strains and integrate them into a traditional classification system: 1) they belong to truly unknown species lacking a scientific name; or 2) the sequencing of taxonomic marker genes from authentically validated and named species still lags behind the sequencing of new collected fungal endophytes and fungal environmental DNA. The former reason seems surprising considering the widespread distribution of the fungus and its ease of culturing, while the latter is a well-acknowledged scientific knowledge gap [28], [30], [31], [32].

Recently, we obtained three ascomata-derived cultures of a fungus resembling *H. investiens* collected in Martinique. Sequencing and database searching of the ITS region of these strains suggested conspecificity with the NAs-producing *Nodulisporium* sp. strains. Therefore, we tested the hypothesis that these collections might represent the sexual state of the NAs-producing *Nodulisporium* sp. by multi-gene phylogeny and by chemical analysis of key secondary metabolites, including *in-vitro* production of NAs. We established that the ascomata-derived *Hypoxylon* strains produced a spectrum of NAs and were genetically congruent with the monophyletic lineage of pantropical *Nodulisporium* endophytes. A new species, *H. pulicicidum*, is proposed to accommodate this widespread cryptic organism.

More than three decades of endophyte research, partly driven by searches for potentially new chemical and biological applications, has revealed that endophytic fungi are hyperdiverse and the gap between their discovery and taxonomic community’s ability to name and classify them is ever widening [31], [33], [34]. The ability to link cryptic endophyte life cycle states is crucial to the understanding of their dispersal and colonization of plants, the differences between beneficial and pathogenic fungi, and the evolutionary pressures that select for the biosynthesis of insecticidal and other bioactive metabolites. Furthermore, establishing a complete life cycle consolidates disparate observations of an organism, whether from environmental sequences or from specimens, resulting in a holistic species concept that is critical to the assessment of the dimensions of fungal diversity and nomenclatural stability [30], [35].

## Results

### DNA Sequence Data and Alignments

The amplification of ITS1-5.8S-ITS2, α-actin and β-tubulin gene fragments of ascomata-derived NAs-producing strains CBS 122622 and MUCL 49879 and a degenerate ascomata-derived strain MUCL 53764, rendered equal size amplification fragments of 661, 272 and 1518 bps respectively. The fragment sizes coincided with those reported from strains MF6263 and MF6321. All these strains had 5 degenerate variant repeat motifs repeated in tandem within the ITS1 gene fragment [36]. DNA sequence comparisons showed that the percentage of similarity among the sequence of the ITS region of these isolates varied from 98 to 99%, with strains MF6263 and MUCL 49879 being the most similar. When the tandem repeat motifs were excised from the ITS1 regions, the similarity of both strains with the remaining NAs-producing strains varied from 95 to 99% and from 88 to 89% with strains from *H. investiens* (CBS 118185, CBS 188183), the phylogenetically closest species observed until now. The β-tubulin gene sequence was identical in ascomata-derived isolates CBS 122622 and MUCL 49879, and the similarity among the remaining NAs-producing strains ranged from 94 to 98%, while the two strains of *H. investiens* were 87 to 88% similar to ascomata-derived isolates CBS 122622 and MUCL 49879. The similarity of the α-actin gene fragment among the ascomata-derived isolates was 97%, and ranged from 93 to 97% with the remaining nodulisporic acid producers. As previously reported [22], α-actin gene fragments were the most variable of the marker regions we examined among the conspecific NAs-producing strains.

### Integration of Multigene Phylogenies of Reference Strains and NAs-producing Strains and Other Xylariaceae

**Figure 2:**
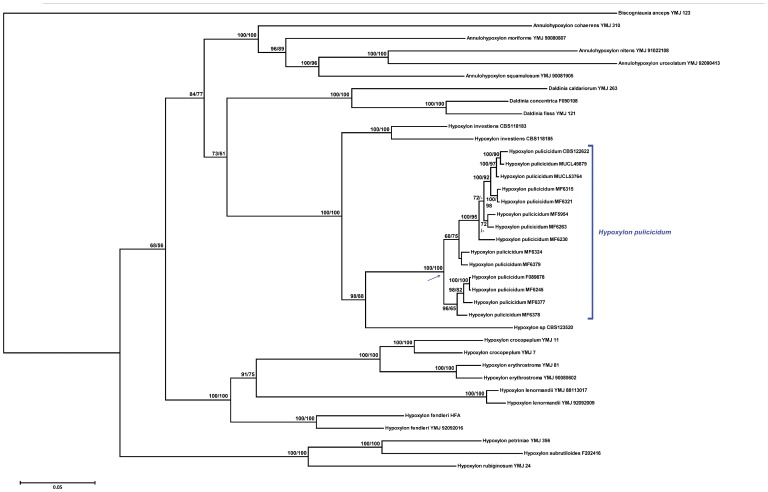
Phylogenetic tree of *Hypoxylon pulicicidum* and related species of the Xylariaceae inferred from Bayesian analysis of a combined gene genealogy of α-actin, β-tubulin and ITS-5.8S-ITS2 genes. *Biscogniauxia anceps* was designated the outgroup. Clade probability values/maximum likelihood bootstrap values are indicated respectively at the branches. Values <50 are designated by -. Bar represents 10 changes.

The new strains, MUCL 49879 and CBS 122622, derived from the ascomata of specimen MJF 07147 and MUCL 53764 derived from specimen CLL 0727 clustered within the clade of NAs-producing endophytes in the combined phylogeny of the three genes (Fig. 2) and in each single α-actin and β-tubulin gene-analyses (Fig. S1A, B). In each case, the NAs-producing strains and the Martinique ascomata-derived strains formed a cohesive clade with high credibility support in each of the each of the combined and individual gene phylogenies (Fig. 2, Fig. S1). Sequence similarity searches of strains in our collections also recognized an unidentified endophyte strain from French Guiana (F-089878, Table 1) as a member of the NAs-producing clade. Similar to MUCL 53764, F-089878 exhibited the phenotype of a degenerated strain; both strains produced only lightly pigmented aerial mycelium, failed to produce conidia on any medium, and under conditions favorable for NAs production they did not produce NAs.

**Table 1:** Living strains examined and their sequences. GenBank accession codes for previous and new sequences used during this work.

Specimen and strain designations^a^	Fungal species	Substratum or host	Geographic origin	ITS	ß-tubulin	α-actin	Comments^b^	References
**MF5954, ATCC 74245**	*H. pulicicidum,*	*Stem of dried herbarium specimen of Bontia daphnoides*	Hawaii, USA	AF201753	FJ185291	FJ185270	Original NAs producing strain, mutants used for large-scale production	[4], [20]
**MF6263**	*H. pulicicidum*	Twig of unknown plant	Colombia	AF201749	FJ185292	FJ185273	Produces NAs	[20]
**MF6321**	*H. pulicicidum*	Unidentified forest litter	Mauritius	AF201751	FJ185297	FJ185275	Produces NAs	[20]
**MF6315**	*H. pulicicidum*	Litter of *Coffea* sp.	Mauritius	AF201754	FJ185293	FJ185274	Produces NAs	[20]
**MUCL 49879, CBS 122622 (ex-holotype strains)**	*H. pulicicidum*	Derived from MJF 07147(holotype), rotten wood	Martinique	JX183075, JX183076	JX183072, JX183074	JX183069, JX183071	Produces NAs	This report
**MUCL 53764**	*H. pulicicidum*	Derived from CLL0727. Rotten wood	Martinique	JX183077	JX183073	JX183070	Degenerate strain, NAs not detected	This report
**MF6230**	*H. pulicicidum*	Horse dung	French Polynesia, Marquesas Islands	AF201752	FJ185288	FJ185271	Produces NAs	[20]
**MF6245**	*H. pulicicidum*	Fruiticose lichen, *Usnea* sp.	Puerto Rico	AF201756	FJ185296	FJ185272	Produces NAs	[20]
**F-089878**	*H. pulicicidum*	Unidentified plant	French Guiana	FJ185305	FJ185294	FJ185268	Degenerate strain, NAs not detected	This report
**MF6378**	*H. pulicicidum*	Twig, *Anacardium occidentale*	Equatorial Guinea	AF201760	FJ185295	FJ185278	Produces NAs	[20]
**MF6379**	*H. pulicicidum*	Twig, *Dorstenia elliptica*	Equatorial Guinea	AF201761	FJ185290	FJ185279	Produces NAs	[20]
**MF6377**	*H. pulicicidum*	Twig, *Scaevola plumeri*	Equatorial Guinea	AF201757	FJ185287	FJ185277	Produces NAs	[20]
**MF6324**	*H. pulicicidum*	Unidentified forest litter	Peru	AF201759	FJ185289	FJ185276	Produces NAs	[20]
**CBS 118183**	*H. investiens*	Derived from TL-6003	Malaysia	FJ185307	FJ185298	FJ185265	NAs not detected	[22]
**MUCL 53758**	*H. investiens*	Derived from CLL10011	French Guiana	Not sequenced	Not sequenced	Not sequenced	NAs not detected	This report

a Strains designated with MF are maintained at Merck Research Laboratories and at the Fundación MEDINA. Strains designated with F-xxxxx are maintained at the Fundación MEDINA.

b Production of nodulisporic acids (NAs) were observed in previous referenced studies and/or this report.

In each phylogenetic tree, either with combined or individual genes, strains of *H*. *investiens* and an unidentified endophyte strain from Mexico, *Hypoxylon* sp. CBS 123520, formed sister lineages (Fig. 2, Fig. S1). Related taxa of the Xylariaceae, e.g., the *Hypoxylon* sensu stricto group, *Annulohypoxylon* and *Daldinia* formed distant lineages. As previously observed [20], [21], [22], the phylogenetic analysis grouped the NAs producing strains into a well-supported clade (100% clade probability and bootstrap support). However, the strains consistently segregated into two or three subgroups as a result in differences in the length of their ITS1 sequences.

### ITS Phylogeny of Endophyte and Environmental Sequences

**Figure 3:**
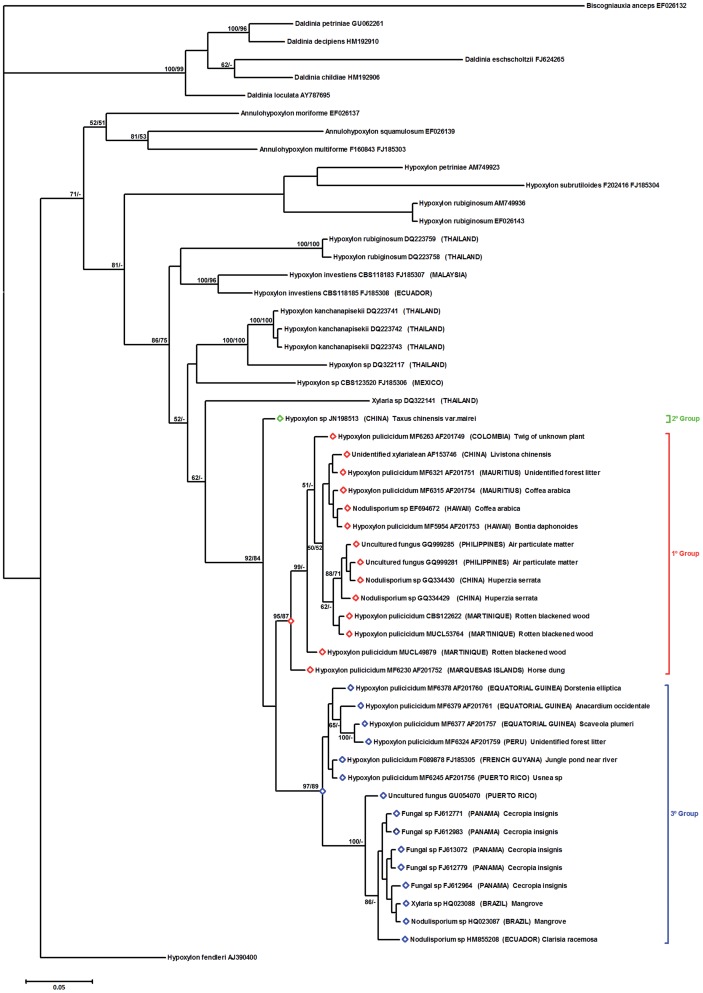
Phylogenetic tree of living strains of *Hypoxylon pulicicidum* and related environmental strains and sequences deposited in GenBank inferred from Bayesian analysis of filtered ITS sequences. *Hypoxylon fendleri* was designated the outgroup. Clade probability values/maximum likelihood bootstrap values are indicated respectively at the branches. Values <50 are designated by -. Bar represents 10 changes. *Hypoxylon pulicicidum* ITS1 sequence groups are identified as group1 with long repeated motifs, group 2 with intermediate repeated motifs, and group 3 with short repeated motifs. ITS1 base pair lengths are listed at the far right.

Homology sequences searches for ITS sequences with high similarity to those of the NAs-producing fungi detected multiple highly homologous sequences. A total of 29 sequences fell within the NAs-producing fungi clade with significant statistical support and were considered to either be conspecific or belonging to a highly related sister lineage (Fig. 3). Researchers have found an isolate from *Coffea arabica* in Hawaii [37] with an ITS sequence (EF694672) identical to the original MRL isolate MF5954 (Table 1). Plant-associated isolates >98% similar to one or more of the previously characterized NAs-producing strains have also been found in seeds of *Cecropia insignis* after soil incubation for five months in Panama [38], from the forest tree, *Clarisia racemosa,* in Ecuador (HM855208), from a palm, *Livistona chinensis,* in Hong Kong [39] and the clubmoss, *Huperzia serrata*, in Hainan Provence, China [40]. Brazilian strains HQ023088 and HQ023087, identified as *Xylaria* sp. and *Nodulisporium* sp., respectively were isolated from unidentified mangroves in São Paulo state. Another sequence from a strain isolated from *Taxus chinensis* var. *mairei* in China (*Hypoxylon* sp., JN198513, Fig. 3) exhibited an intraspecific ITS repeat tandem motif not previously observed. Depending on the alignment method, retaining or excising the repeated motifs, this strain fell within the NAs group or was a sister group, indicating the existence of yet other closely related populations. Although near matches for sequences from subtropical and tropical endophytes were expected, more surprising were near matches for three sequences derived from biological particulates from air samples taken on a ship off the coast of the southern Philippines (GQ999285, GQ999281) and from Puerto Rico (GU054070) attributed to an uncultured fungus [41] (Fig. 3). The sequences were obtained from DNA extracted from aerosol particulate samples and amplified with fungal specific ITS primers; amplicons were then cloned and sequenced [41]. Trapping airborne propagules of the NAs-producing fungi signifies that these fungi can be carried between land masses by long distance aerial dispersal.

Alignment of ITS1-5.8S-ITS2 gene sequences revealed that two different populations with ITS1 tandem repeat motifs varying in the number of repeats existed, and a possible third group that may be a sister lineage (Fig. 3). The first group encompassed sequences from strains or biological samples collected in tropical Africa and South America (Atlantic zone) with ITS sequence similarities from 97 to 100% and three tandem repetitions of the variant repeat in the ITS1 region. The second group comprised strains and sequences originating primarily in the tropical Asia-Pacific zone, with the exception of the ascomata-derived strains CBS 122622, MUCL 49879 and MUCL 53764 from Martinique. Their sequences had 4 or 5 repetitions of the variant repeat. The sequence of the 4th variant repeat was identical in the isolates with the largest ITS1, indicating that deletion, rather than insertion, caused the size variability of the ITS1. The deletion of this central tandem repeat motif seems to have occurred independently in different geographical locations, as inferred from the sequence homology between strains MF6321 and MF6315 from Mauritius [20], MF5954 and EF694672 from Hawaii and GQ334429 and GQ334430 from China. A possible third group, which might be interpreted as a potential sister lineage, consisted of a single sequence designated as a *Hypoxylon* sp., an endophyte isolated from *Taxus chinensis* from China (JN198513, Fig. 3).

### Production of Nodulisporic Acids

The hypothesis that the ascomata-derived strains from Martinique would produce NAs was first tested by growing the strains MUCL 49879 and MF5954 on six media (BRFT, CYS80, DEF2, LSFM, MV8, MMK2) for 14 d. Acetone extracts from each fermentation were screened by LC-MS for combinations of ion fragmentation patterns, retention times, and UV signals that matched signals from authentic samples of NAs A, C_2_, D_1_ and F (Fig. S2). Strain MUCL 49879 was positive for NA A on medium LSFM while MF5954 was positive for NA A on BRFT, LSFM, MV8 and MMK2. The screen for NAs production was repeated with three strains of *H. investiens*, the two sister ascomata-derived strains MUCL 49879 and CBS122662, the degenerate ascomata-derived strain MUCL53764, degenerate endophyte strain F-089,878, and an expanded panel of NAs-producing endophytes (MF5954, MF6230, MF6263, MF6324, MF6245, MF6321, MF6315, MF6378, MF6379) grown in a matrix of two fermentation media favoring NAs production (LSFM, MMK2) at two different harvest times (14, 21 d). Strains MUCL 49879, CBS 122662, MF5954, MF6230, MF6321, MF6315, MF6378, and MF6379 were positive for at least one NA on at least one medium-harvest time combination (Table 2). The medium LSFM was the most effective medium for eliciting NAs production in most cases.

**Table 2:** Preliminary identification of nodulisporic acids in fermentation extracts of strains of *Hypoxylon investiens* and *H. pulicicidum* by LC-MS database matching.

Identification	Strain^a^	Medium^b^	Nodulisporic acid analogs present^C^			
			A	C_2_	D_1_	F
*H. pulicicidum* degenerate endophyte strain	F-089,878	LSFM	−	−	−	−
		MMK2	−	−	−	−
*H. investiens*	CBS 118183	LSFM	−	−	−	−
		MMK2	−	−	−	−
*H. investiens*	CBS 118185	LSFM	−	−	−	−
		MMK2	−	−	−	−
*H. investiens*	MUCL 53758	LSFM	−	−	−	−
		MMK2	−	−	−	−
*H. pulicicidum* degenerate teleomorph strain	MUCL 53764	LSFM	−	−	−	−
		MMK2	−	−	−	−
*H. pulicicidum* teleomorph strain	MUCL 49879	LSFM	+	−	+	−
		MMK2	−	−	+	−
*H. pulicicidum* teleomorph strain	CBS 122622	LSFM	+	−	+	+
		MMK2	−	−	+	−
*H. pulicicidum* endophyte strain	MF5954	LSFM	+		+	+
		MMK2				+
*H. pulicicidum* endophyte strain	MF6230	LSFM	−	−	+	−
		MMK2	−	−	−	−
*H. pulicicidum* endophyte strain	MF6263	LSFM	−	−	−	−
		MMK2	−	−	−	−
*H. pulicicidum* endophyte strain	MF6264	LSFM	−	−	−	−
		MMK2	−	−	−	−
*H. pulicicidum* endophyte strain	MF6321	LSFM	+	−	+	−
		MMK2	−	−	+	−
*H. pulicicidum* endophyte strain	MF6315	LSFM	−	+	−	−
		MMK2	−	+	+	−
*H. pulicicidum* endophyte strain	MF6379	LSFM	−	+	+	−
		MMK2	−	−	−	−
*H. pulicicidum* endophyte strain	MF6378	LSFM	+	−	+	−
		MMK2	−	−	−	−

a See Table 1 for strain origins and details.

b See Methods and Material for medium formulations, fermentation and extraction protocols. Either fermentations harvested at 14, 21 d with nodulisporic acids, or both were considered positive.

c Identification based on database matching of ion fragmentation patterns from authentic nodulisporic acids chromatographed fermentation extracts.

A subset of extracts that were strongly positive for NAs, including extracts from the sister ascomata-derived strains, was selected and further concentrated to about 5× relative to the original fermentation volume, chromatographed on an identical LC system and analyzed by ESI-TOF-MS to identify and verify the presence of NAs (Table 3). Patterns of NAs production varied across the set, and most of the known NAs were detected in at least one or more of the strains, and the retention times for individual ions were similar across all samples (Table 3). Ions corresponding to the exact masses of NAs A, A_1_, A_2_, C, C_1_, D_1_, and D_2_ were observed in at least one of the extracts from the two sister ascomata-derived strains (Table 3). The sets of ions from the Martinique strains were essentially identical to NAs detected from those of the endophytic strains. Thus, the two strains cultured from the Martinique *Hypoxylon* specimen MJF 0714 produced a spectrum of NAs similar to that of the *Nodulisporium* endophytes discovered at MRL, while on the other hand, NAs could not be detected in authentic strains of *H. investiens*.

**Table 3:** Detection and distribution of nodulisporic acid analogs by liquid chromatography high-resolution mass spectrometry among fermentation extracts of selected endophytic and teleomorph-derived strains of *Hypoxylon pulicicidum*.

Stain and country of origin*	Medium and days fermented*	Nodulisporic acid analogs and their calculated *m/z* values											
			A	A1	A2	B2	C	C1	C2	D1	D2	E	F
		Calc. *m/z*+H	680.3946	696.3895	714.4000	700.4208	668.4310	684.4259	702.4364	600.3684	618.3789	572.4098	436.2846
		Calc. *m/z*+H-H2O	662.3840	678.3789	696.3895	682.4102	650.4204	666.4153	684.4259	582.3578	600.3684	554.3993	418.2741
		Calc. *m/z* +Na	702.3765	718.3714	736.3820	724.4184	690.4129	706.4078	724.4184	606.3554	640.3609	594.3918	458.2666
			Nodulisporic acid analogs and their observed *m/z* values										
CBS 122622 Martinique	LSFM 21 d	RT (min)		6.05	5.56		6.93	6.05		6.14	5.11		
		Meas. *m/z* +H		696.3902			668.4310	684.4251		600.3686	618.3777		
		error in ppm		1.0			0.1	−1.1		0.4	−2.0		
		Meas. *m/z* +H-H2O		678.3789	696.3897		650.4192	666.4141		582.3579	600.3685		
		error in ppm		0.0	0.3		−1.8	−1.8		0.2	0.2		
		Meas. *m/z* +Na			736.3828								
		error in ppm			1.1								
MUCL 49879 Martinique	LSFM 21 d	RT (min)	5.87	6.06	5.57		6.88			6.13	5.68		
		Meas. *m/z* +H	680.3919	696.3893			668.4309			600.3693	618.3787		
		error in ppm	−3.9	−0.3			−0.1			1.6	−0.3		
		Meas. *m/z*+H-H2O	662.3839	678.3793	696.3897		650.4197			582.3584	600.3686		
		error in ppm	−0.2	0.6	0.3		−1.1			1.1	0.4		
		Meas. *m/z* +Na			736.3829								
		error in ppm			1.2								
MF 6315 Mauritius	MMK2 14 d	RT (min)							5.67	6.17			
		Meas. *m/z* +H							702.4355	600.3686			
		error in ppm							−1.3	0.4			
		Meas. *m/z* +H-H2O							684.4268	582.3591			
		error in ppm							1.4	2.3			
MF 6379 Equatorial Guinea	LSFM 14 d	RT (min)				5.59	6.85			6.20			
		Meas. *m/z* +H				700.4217	668.4297			600.3698			
		error in ppm				1.3	−1.9			2.4			
		Meas. *m/z* +H-H2O				682.4112	650.4185			582.3578			
		error in ppm				1.4	−2.9			0.0			
MF 6378 Equatorial Guinea	LSFM 21 d	RT (min)	5.86		5.55		6.88			6.14			
		Meas. *m/z* +H	680.3944				668.4317			600.3701			
		error in ppm	−0.2				1.1			2.9			
		Meas. *m/z* +H-H2O	662.3843		696.3901		650.4182			582.3585			
		error in ppm	0.5		0.9		−3.4			1.2			
		Meas. *m/z* +Na			736.3799								
		error in ppm			−2.8								
MF6230 Marquesas Islands	LSFM 21 d	RT (min)		6.03	5.55					6.18			
		Meas. *m/z* +H		696.3911						600.3687			
		error in ppm		2.3						0.6			
		Meas. *m/z* +H-H2O		678.3792	696.3899					582.3577			
		error in ppm		0.4	0.6					−0.1			
		Meas. *m/z* +Na			736.3828								
		error in ppm			1.1								
MF 6321 Mauritius	LSFM 21 d	RT (min)	5.96	6.14	5.57		6.91			6.16		6.96	5.32
		Meas. *m/z* +H		696.3891			668.4302			600.3688		572.4089	436.2837
		error in ppm		−0.5			−1.1			0.7		−1.6	−2.1
		Meas. *m/z* +H-H2O	662.3835	678.3776	696.3892		650.4194			582.3579			
		error in ppm	−0.8	−1.9	−0.4		−1.5			0.2			
		Meas. *m/z* +Na	702.3754	718.37	736.3824							594.3904	458.2679
		error in ppm	−1.6	−2.0	0.6							−2.3	2.9

* SeeTable 1 for strain details and Methods and Material for description of fermentation media and protocols.

Calculated (Calc.) *m/z* for the known nodulisporic acids and their dehydrated and Na^+^ adducts are given above. For each strain, the retention times (RT), measured (Meas.) m/z and error in ppm are given for each detected nodulisporic acid ion and its dehydrated and Na+ adducts, respectively.

### Taxonomy

**Figure 4:**
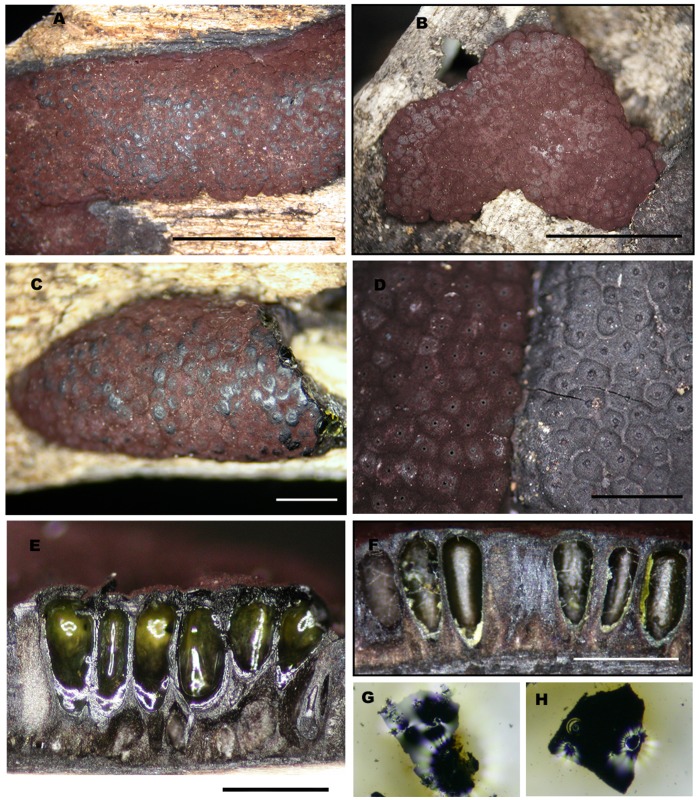
*Hypoxylon pulicicidum.* A, C, F, G: CLL0727; B, D, E, H: Holotype MJF 07147. A-C. Stromata. d. Close up of stromatal surfaces of a purplish mature stroma (left) and a blackish overmature stroma with slightly papillate ostioles (right). E, F. Stromata in vertical section showing perithecia with green contents (rehydrated in e). G, H. KOH-extractable pigments. Scale bar A-C = 5 mm; D-F 1 mm.

***Hypoxylon pulicicidum*** **J. Fourn., Polishook & Bills,** ***sp. nov.*** **Figs. 4** **,** **5** **,** **6** **.**

Mycobank: MB800260.

Etymology: *pulicicidum*, flea killing.

**Figure 5:**
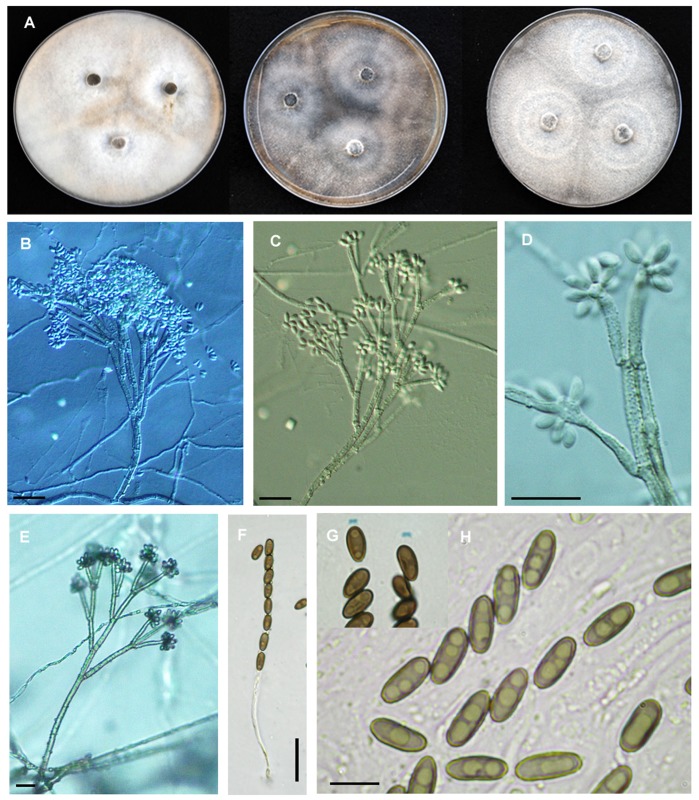
Cultures and microscopic features of *Hypoxylon pulicicidum*. A. MUCL 49879, from left to right, 14-d-old cultures on oatmeal agar, malt-yeast extract agar, 2% malt agar. B. MUCL 49879, conidiophores from SNA. C. MUCL 49879, conidiophores from SNA. D. MUCL 49879, conidiogenous cells and conidia from SNA. E. MUCL 49879, Aerial view of conidiophore from SNA. F. Ascus mounted in 1% SDS (MJF 07147). G. Ascus apical apparatus stained in Melzer’s reagent. H. Ascospores mounted in water (CLL 0727). Scale bar: F. 20 µm; H. 10 µm.

**Figure 6:**
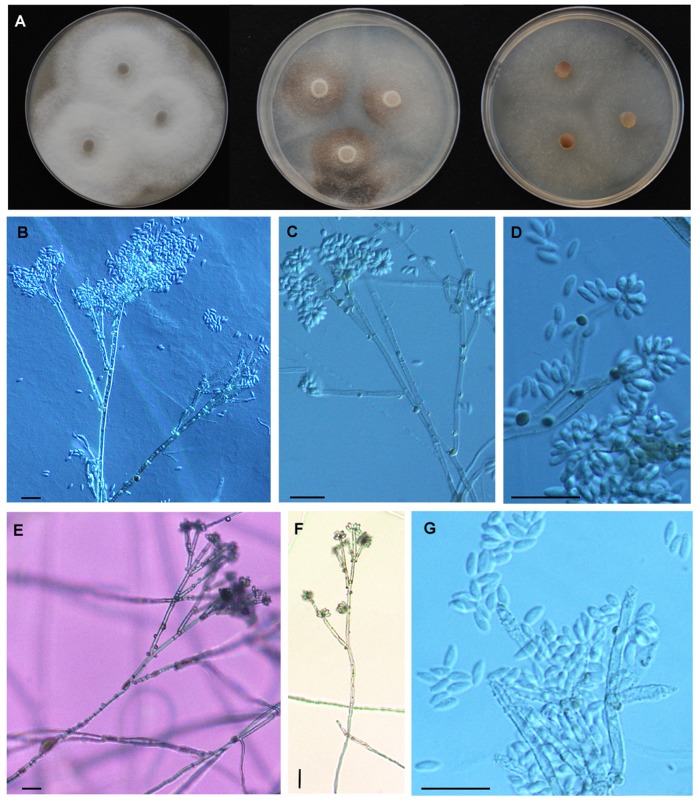
Cultures and microscopic features of *Hypoxylon pulicicidum*, MF5954. A. from left to right, 14-d-old cultures on oatmeal agar, malt-yeast extract agar, 2% malt agar. B. Conidiophore from SNA. C. Conidiophore from SNA, note dark pigment granules. D. Conidiogenous cells and conidia from SNA, note dark pigment granules. E. Aerial view of conidiophore from SNA. F. Aerial view of conidiophore from SNA. G. Conidiogenous cells and conidia from SNA.

#### Diagnosis

A *Hypoxylon* species with stromata similar to *H. investiens*, but differing in its stromatal pigment granules that release a pale green KOH-soluble pigment, rather than a dark olive pigment, the absence of daldinone A in its stromata, and with distinct ribosomal DNA, α-actin and β-tubulin sequences. Vegetative mycelia widespread in tropical plants and plant debris. Cultured mycelia usually with a fragrant and sweet odor. Usually producing nodulisporic acids in culture. Holotype MJF 07147 (LIP).

**Stromata** irregularly effused-pulvinate to elongate (Fig. 4A–D), 7–50×3–22×1–1.4 mm, often growing beside and over old black depauperate stromata, with faintly exposed discoid perithecial contours (Fig. 4A–D); surface pruinose, Dark Vinaceous (82) to Brown Vinaceous (84), subsurface blackish, composed of weakly carbonaceous tissue and inconspicuous brownish black granules yielding dilute Olivaceous Buff (89) pigments in 10% KOH (Figs. 4G, 5H); subperithecial tissue 0.2–0.5 mm thick, fibrous, grey brown to blackish, seated on a thin black line (Fig. 5E–F). **Perithecia** lanceolate, 0.7–0.9×0.3–0.35 mm, with perithecial contents Citrine Green (67) (Fig. 5E–F). **Ostioles** umbilicate, faintly papillate in old black stromata (Fig. 4D).

**Asci** few, 90–110 µm (Fig. 5G–H), the spore-bearing parts 55–65×4.5–5 µm, the stipes 30–45 µm, originating in unilateral spicate arrangement from long ascogenous hyphae, with amyloid apical apparatus discoid, 0.8×1.7–2 µm. **Ascospores** 7.5–9.4×3.2–4.2 µm (M = 8.6×3.5 µm, n = 30), ellipsoid equilateral with broadly rounded ends to oblong, pale brown (Fig. 5G–H), uniseriate in the ascus, with faint germ slit spore-length to slightly less than spore-length (Fig. 5H). Perispore indehiscent in 10% KOH; epispore smooth.

**Colonies** (22°C, 2 wk, Figs. 5A, 6A) on OA attaining 38–45 mm, at first velvety to felty, finally wooly to hispid, azonate, margins diffuse to fimbriate, pale yellow brown to greenish brown (4F6), finally dull grayish brown (5E3, 5F3), dull reddish gray (7E4-3, 7F4-3), occasionally with dark brownish black exudates in age, sporulation scattered on aerial hyphae after 2 to 3 wk, reverse dull yellowish gray (5E2, 5F2) to grayish red brown (8F3-2). On MEA attaining 37–45 mm, felty, faintly zonate, margins fimbriate to submerged, pale gray to dull reddish gray (8E3-1, 8F3-1), finally dull gray brown, scant sporulation on the aerial hyphae, reverse deep reddish black (9F3-2) to black. On YM attaining 35–43 mm, appressed to felty, with radial hyphal strands running across the agar surface, pigmentation similar to MEA, scant sporulation on aerial hyphae, reverse with strong black mottling. On SNA attaining 30–40 mm, and 14 mm before agar desiccated, mostly submerged, hyaline at first but in age forming olive brown to grayish brown hyphal strand radiating from central inoculation point, with pale buff sporulation evident on agar surface, especially along axes of hyphal strands. Agar plugs of MUCL 49879 incubated at 37°C on YM only developed scant hyphae from the agar plug, while MF5954 grew about 3–4 mm. **Conidiophores** (Figs. 5B–E, 6B–F) on SNA arising from repent hyphae on agar surface, often flowing or concentrated along axes of hyphal strands, erect, up to 300 µm tall, 4–7 µm wide at the base, septate, hyaline to olive brown or grayish brown in 5% KOH, finely roughened, with scattered brownish gray pigment granules, slightly thick walled at the base, with up to 4 levels of branches, with first primary branch, between one- to two-thirds up the main axis, with 2–4, but usually 3, terminal conidiogenous cells, rarely with intercalary conidiogenesis. **Conidiogenous cells** (Figs. 5D, 6D, 6G) usually arising from terminal cells, rarely with swollen intercalary loci, bearing a cluster of holoblastically produced conidia, determinate, cylindrical, mostly with a slightly clavate to inflated apex, finely roughened, bearing finely denticulate secession scars at and slightly below the apex, 10–22 µm×2–4 µm. **Conidia** hyaline, white to buff in mass, smooth, ovoid to ellipsoid, with truncated basal scar, 4–7 µm×2–3 µm, usually with a small guttule at each end, failing to germinate or only forming aborted germ tubes in agar culture (Fig. S3).

#### Distribution of ascomata

**Martinique:** Prêcheur, Anse Lévrier, coastal mesophilic forest, Sept. 2003, on rotten wood, Christian Lechat, CLL0727 ex herb. J. Fournier (MUCL 53764); Prêcheur, Anse Couleuvre, coastal mesophilic forest, 27 Aug. 2007, on rotten blackened wood, Jacques Fournier MJF 07147 (holotype LIP), ex-holotype cultures MUCL 49879, CBS 122622, obtained from two independent isolations from centrum contents and therefore varying slightly in their ITS nrDNA sequences.

#### Distribution of endophytic state

Pantropical**, s**ee strains listed in Table 1, in Polishook *et al.* [20], and environmental and endophyte sequences listed in Fig. 3.

#### Comments

The above description is based primarily on the two different ex-holotype cultures derived from MJF 07147 (MUCL 49879 and CBS 122622) and Merck’s original Hawaiian strain (MF5954) which was grown in parallel on the same media to better understand whether the strains differed phenotypically (Fig. 6). The conidiophores of MUCL 49879 and MF5954 are typical of the *Nodulisporium* type, but have more terminal conidiogenous cells compared to most other Xylariaceae conidial states. Unlike *H. pulicicidum*, the conidiphores of *H. investiens* have been described as *Periconiella*-like [25]. Its conidiogenesis cells tend to arise from the main conidiophores axis, often having terminal conidiogeneous cells in whorls, and its conidiophores are no more than 120 µm tall. MUCL 49879 shares the basic suite of characters of the NAs-producing *Nodulisporium* strains, an aromatic sweet odor, minutely roughened conidiophores with solid droplets of a melanin-like brown material on the conidiophores and aerial hyphae (Figs. 5, 6), conidiogenesis restricted to the apical regions of the terminal conidiogenous cells, a dark brown soluble pigment in agar medium and fermentation broths, especially when supplemented with complex nitrogen supplements like yeast extract. When MUCL 49879 was compared side-by-side to the Hawaiian NA-producing strain MF5954 on the same medium (SNA, Figs. 5, 6)), some subtle phenotypic differences were evident. In MUCL 49879, the terminal conidiogenesis cells were slightly more inflated, the conidiophores branching pattern slightly was more regular and symmetrical, pigment granules were more finely dispersed on the conidiophores, and its sporulation was less profuse than that of MF5954. When grown at 37°C, MF5954 attained 20 mm radial growth on cornmeal agar before the agar desiccated, while MUCL 49879 only produced a few hyphae from the agar plug on cornmeal agar, although some growth was evident on SNA. In this study, we did not observe that the NAs-producing strains grew faster at 37°C than at 22–28°C [4], [20], [22]. However, limited growth at 37°C was evident. As observed previously [20], the conidia from cultures of MF5954 formed germ tubes but failed to develop more than a single cell, whereas in MUCL 49879 conidia completely failed to germinate (Fig. S3). Whether conidia formed in nature behave similarly remains unknown, but such behavior in nature would call into the question the role the conidia as dispersal propagules. Nevertheless, we observed that hyphal and conidiophore fragments germinate readily when transferred from one medium to another (Fig. S3).

### Stromatal Secondary Metabolites

**Figure 7:**
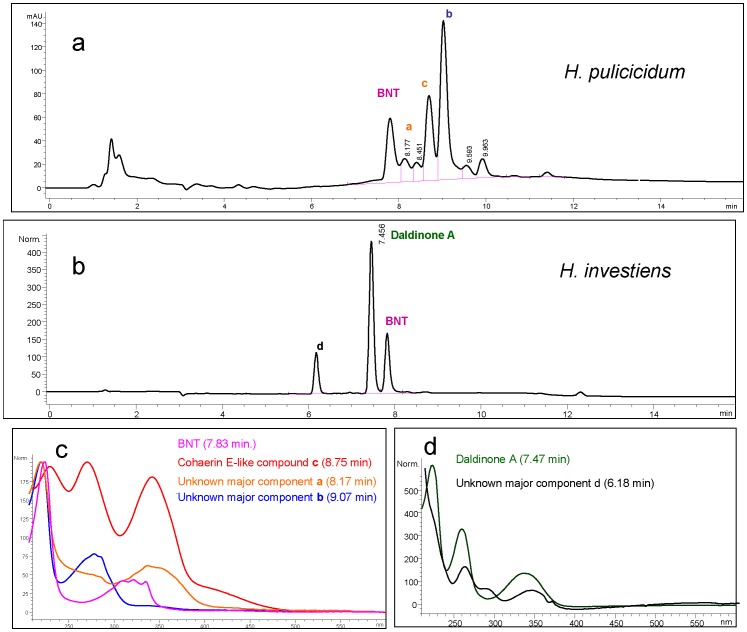
HPLC-UV chromatograms of *Hypoxlyon pulicicidum* and *H. investiens*. 9a. HPLC-UV chromatograms of stromatal methanol extracts of the *H. pulicicidum* (MJF 07147, Holotype). 9b. HPLC-UV chromatograms of stromatal methanol extracts of specimen of *H. investiens* s. str. (material from Taiwan, see Fig. S4). 9c. UV spectra of BNT and unknown components from *H. pulicicidum*. The unknown compound B in *H. pulicicidum* has a MW of 506 DA. 9d. UV spectra of components from *H. investiens* including daldinone A and another unknown compound D.

The HPLC profiles of the stromatal methanol extracts of *H. pulicicidum* differed from those of typical *H. investiens* (Fig. 7A vs. 7B). The extracts of *H. investiens* stromata mainly contained 1,1′-binaphthalene-4,4′-5,5′-tetrol (BNT), a metabolite ubiquitous in hypoxyoid Xylariaceae [42], [43] and its oxidized derivative, daldinone A (Fig. 7B, D). The new species contained BNT and additional components undetected in *H. investiens*. The major components of the crude extract of *H. pulicicidum* were searched against a HPLC-DAD/MS library [44] linked to a comprehensive database of Xylariaceae metabolites compiled during the past ten years [26]. No matches were found for the *H. pulicicidum* components; NAs were not found in the stromata. However, one of the apparently specific compounds (c) found in *H. pulicicidum* closely resembled cohaerin E, previously isolated from *Annulohypoxylon cohaerens* [45].

Despite the fact that peak b in *H. pulicicidum* (Fig. 7A) resembled cohaerin E in both its UV-visible chromophore and its retention time in a standardized HPLC system, the ESI-MS analysis revealed that the molecular mass of compound was four Da less than cohaerin E (MW 462 vs. 466, data not shown). Possibly this metabolite could be a new cohaerin derivative with two additional double bonds. However, characterization of its structure by NMR and HRMS will need to await larger collections of *H. pulicicidum* stromata in order to attempt preparative isolation work. Significantly, daldinone A, a metabolite found in all authentic specimens of *H. investiens* examined to date, was not detected in *H. pulicicidum* (Fig. 7A vs. 7B). Furthermore, cohaerins were thought to be the coloring matter responsible for stromatal pigments of *A. cohaerens*. Therefore, the superficially similar KOH-extractable pigments of *H. pulicicidum* and *H investiens* were due to entirely different molecules, thus reinforcing the recognition of *H. pulicicidum* as a separate taxon.

## Discussion

### Segregation of *H. investiens* and Related Species

*Hypoxylon pulicicidum* is externally indistinguishable from typical *H. investiens* (Fig. S4) with which it has in common effused-pulvinate stromata with vinaceous brown surface, slightly exposed perithecial contours that are discoid-flattened and tubular to lanceolate perithecia. Furthermore, both have similar ellipsoid-equilateral ascospores with a non-dehiscing perispore in 10% KOH.

Besides its ability to produce NAs, its different conidiophore structure and its lack of daldinone A in its stroma, this new species can be distinguished from typical *H. investiens* by its different KOH-extractable pigments and an unusual greenish color of the perithecial contents, best viewed when rehydrated (Fig. 4G, H). It is noteworthy that in both collections studied, although the stromata seemed mature in all respects, very few asci had differentiated. Outdoor incubation of specimen MJF 07147 during 10 days in Martinique failed to promote any further maturation. Possibly, ascogenesis in this species is delayed or weak, perhaps a condition contributing to the apparent rareness of its ascomata and shift towards dominance of its endophytic state [20]. Microscopically, its ascospores appeared slightly smaller and narrower than those of *H. investiens* (Fig. S4), and with a less conspicuous germ slit. Based solely on ascomata morphology, this new species might be accommodated in the fairly broad concept of *H. investiens* as assessed by Ju & Rogers [25] which most likely encompasses additional cryptic species, e.g., *Hypoxylon* sp. CBS 123520 and other collections (MS, JF unpublished data). *Hypoxylon pulicicidum* is one of these cryptic species, and its recognition again demonstrates how a polythetic approach can discriminate among a complex of closely related species.

### Significance of Nodulisporic Acid in the Fungus Life Cycle

Several media were tested for detection of NAs, and LSMF medium was the most consistent for stimulating production of NA A and other minor NAs. Previous work demonstrated that NA A titers from wild type isolates, even after extensive medium optimization studies, rarely surpassed 10 mg/l. The significant yield improvements needed to support semi-synthetic chemical optimization and animal dosing were only achieved after multiple cycles of mutation followed by medium optimization [5], [7]. These observations suggest, that like other fungal metabolites, the biosynthesis of NAs is tightly regulated.

Inheritance and maintenance of an indole diterpene biosynthetic gene cluster would impose a high cost for the fungus [11], [46], and therefore the pathway is likely to contribute to the fitness of the fungus. NAs are relatively facile to produce *in vitro,* at least at low titers when the fungus is grown on a suitable medium. Therefore, it seems reasonable to assume that the NAs biosynthetic cluster is operational at some stage in the fungus’ natural life cycle, and NAs could be produced locally by the mycelium at physiologically relevant concentrations, i.e. at least low ppm concentrations.

Previously, finding endophytic isolates producing NAs was at best haphazard [20], with the only guiding information being a tentative conidial genus and a tropical distribution. Knowing the sexual state now permits tracking of populations of the fungus in tropical forests by location of its persistent stromata on dead wood and will enable investigations of how the fungus disperses to and persists in a wide array of plants, plant debris and dead wood, and whether sexual sporulation is host selective. For example, how patterns of metabolite expression change during stromata maturation have been monitored in *H. fragiforme* in the forest [47]. *Hypoxylon pulicicidum,* a fungus whose life cycle alternates between dead wood and living plants, is easily cultivated in the laboratory and produces a true insecticidal metabolite with extraordinary potency levels and a mode-of-action specifically affecting insect neurology. Therefore, the fungus presents an exceptional opportunity for testing the popular notion that a horizontally-transmitted endophytic fungus has evolved a chemically-mediated arthropod defense system for protecting its own mycelium from mycophagy and its hosts from herbivory [48], [49].

### Host Generalism in Tropical Endophytes

A sexual cycle in *H. pulicicidum* and the concomitant recombination could contribute in part to the sequence variations and polymorphisms evident in the different marker genes and variable patterns of NAs expression among different strains [20], [21]. The sexual cycle may also contribute the fungus securing new wood decay resources. Perithecia-bearing stroma with active ascospore discharge, followed by ascospore germination and nonselective infection of different host plants or lichens [20] is one dispersal scenario and would contribute one mechanism for the organism’s widespread distribution. Similar indiscriminate endophytic infections have been observed for other xylariaceous fungi, incuding *H. fuscum*, *H. fragiforme*, *Biscogniauxia mediterranea B. nummularia* and *Creosphaeria sassafras*, even though their ascomata preferentially develop on wood and bark of specific host plants [26], [27], [29], [50], [51]. Conidia of *H. pulicicidum* form abundantly in culture but have yet to be observed in nature; their role in dispersal or fertilization of mycelia of complementary mating types remains to be characterized. Whether or not the conidia contribute to its dispersal remains unclear, and conidial germination and infection on living plants remains to be tested. We have observed that the fungus remains viable, presumably as vegetative hyphae, for long periods in desiccated plant material, herbivore dung and even in heat-dried herbarium voucher specimens (e.g., MF5954, MF6230, J. Polishook, G. Bills, unpublished data), and conceivably viable hyphae in plant and wood fragments could contribute to its dispersal and eventual colonization of preferred wood resources.

Addition of spurious endophyte and environmental sequences from public databases to our sampling of endophyte cultures and new field specimens significantly expanded our understanding of the distribution of *H. pulicicidum*, including the recognition of a new subpopulation or possible sister lineage in China with a distinct ITS1 tandem repeat motif [20], [21]. The growing availability of environmental sequences encourages data mining and has provided valuable insight into many fungal and microbial lineages. Even though environmental sampling significantly extended our taxon sampling, the void in integration of newly recognized lineages in the environment without unequivocal links to corresponding sequences from authentic specimens remains a major challenge. Sequencing efforts targeting high-quality specimens in herbaria and culture collections and the recollection and authentication of legacy species [52] is the most effective way to take advantage of existing taxonomic knowledge, and therefore to build an interpretive framework for environmental sampling by anchoring environmental and unknown endophyte sequences to meaningful names.

### Nomenclature and Description of New Endophytes

Although the potential novelty of the NAs-producing endophytic isolates was evident from their earliest phylogenetic characterizations, the organisms were not immediately embraced as a new species because of the potential of creating a redundant name for a yet unsequenced *Hypoxylon* species and because of an intuition that these strains would eventually be connected to the sexual state of its life cycle [20]. Restraint in formally naming spurious isolates of endophytic isolates has not always been practiced. In many cases, names have been applied without obtaining multiple isolates and without attempting to understand how their reproductive states might relate to host dispersal, or in the worst cases, without observing any reproductive phenotype. Several instances of this practice have lead to the creation of ill-defined and potentially redundant generic and species names based on vegetative cultures, e.g. *Taxomyces* [53], that now has been considered to be a synonym of *Cladorrhinum* [54]. The case of *H. pulicicidum* and other cryptic fungi once again demonstrate that with persistent collecting and analysis of specimens and cultures, life cycles of organisms that first appear to be spurious endophytes or apparent novel taxa [34], [55], [56], [57] can be elucidated and fully integrated into conventional classifications. Therefore, we believe that the sporulating and vegetative states of many endophyte life cycles eventually can be linked to previously described species and that proposed environmental sequence classifications and specimen based systems eventually will converge [28], [33], [34], [52].

Furthermore, three ITS sequences of *H. pulicicidum* derived from a sensitive PCR and cloning technique applied to air filtrates were classified as an uncultured fungus in Genbank [41], when clearly organisms belonging to the Xylariaceae, fungi that are normally culturable, and comparison with highly similar sequences might have approximated an classification of the sequence at the family level. The ability to anchor disparate sequences of *H. pulicicidum* to a conventional specimen and its cultures illustrates why an unknown endophyte strain should not be assumed to be an undiscovered species and why an unidentifiable environmental sequence should not be assumed to be derived from an uncultured or unculturable fungus. We believe the more rational reaction to such information gaps should be to simply assume that the fungus and its corresponding living specimen or culture has not been sequenced yet or the sequences have not been released to public databases. The historical record of fungal names linked to DNA barcode sequences still lags far behind the current technologies for generation of environmental sequences, while the number of biologists utilizing environment DNA sequences or culturing endophytes far exceeds the few scientists attempting to provide modern fungal descriptions and reconcile phylogenies with existing fungal names. Unless the sequences are from a truly novel lineage, then the more productive and scientifically satisfactory solution would be to thoroughly search for the fungus and its alternate life cycle states and connect authentic specimens and cultures to equivalent sequences.

## Materials and Methods

### Fungal Strains

Fungal strain origins, strain numbers, and identifications are listed in Table 1. Strains were preserved as frozen mycelial discs or mycelial suspensions in 10% glycerol at −80°C and maintained in the collection of Fundación MEDINA, Granada, Spain, unless designated otherwise. Frozen strains were revived by plating frozen agar discs or hyphal fragments onto YM agar (malt extract 10 g; yeast extract 2 g, agar 20 g, 1000 ml distilled H_2_O) at 22°C.

### Fermentation for Detection of Nodulisporic Acids

Mycelia discs from YM agar cultures were used to inoculate 250-ml flasks containing 50 ml of SMYA medium (Difco neopeptone 10 g, maltose 40 g, Difco yeast extract 10 g, agar 4 g, 1000 ml distilled H_2_O). Flasks were incubated in an orbital shaker at 220 rpm for 4 d at 22°C in total darkness. This seed inoculum was used to inoculate fermentations in 250-ml flasks containing 50 ml of liquid media. The media for fermentations were: LSFM (glycerol 18.7 g, glucose 40 g, yeast autolysate (Fluka Biochemika 73145) 5 g, (NH_4_)_2_SO_4_ 2 g, soybean meal 5 g, tomato paste 5 g, sodium citrate 2 g, and 1000 ml distilled H_2_O, and adjusted to pH 7.0); MMK2 (mannitol 40 g, yeast extract 5 g, Murashige & Skoog salts (Sigma-Aldrich M5524) 6.7 g and 1000 ml distilled H_2_O). Recipes for other media used for the initial screening for NAs can be found in [58]. Flasks were inoculated with 2 ml of mycelial suspensions from seed inocula and incubated at 220 rpm for 14 d and 21 d at 22°C in total darkness. All medium×harvest time×strain combinations were repeated at least twice, and unfermented media were submitted for analysis as negative controls.

### Morphology and Culture Studies

Methods for phenotypic comparison of specimens and cultures have been previously detailed [59]. Briefly, perithecial dimensions were determined microscopically at 50–100×. Microscopic features were evaluated in water mounts (brightfield and phase contrast microscopy at 1000× or 1200×). Perispore dehiscence was tested by adding 10% KOH to water mounts. The ascal apical apparatus was visualized with Melzer’s reagent. Ascospores and conidia sizes were based on at least 10 and up to 25 measurements, and are given as the most common range and the extreme values in parentheses. Dimensions for perithecia, asci, ascal apical rings, conidiophores, and conidiogenous cells are based on minimumly five individual measurements. Colors of KOH-extractable pigments were visualized by placing a stromatal fragment including the outer crust in a drop of 10% KOH and observed against a white background. Stromatal pigments contained in tissues dissected from the stromatal crust containing the waxy granules immediately beneath the stromatal surface were evaluated as previously described [25]. Specimens were cultured from centrum contents aseptically microdissected from perithecia, and therefore were presumably multiple spore isolates. Branching patterns and conidiophore types were classified according to the previously described nomenclature [25]. Color codes were determined by comparison with a color chart [60] from stromata, e.g., (30) or a color guide [61] for cultures, e.g. (20E–F4).

In vitro morphology was compared by growing strains on oatmeal agar (Becton Dickenson), 2% malt agar (malt extract 20 g, agar 20 g, distilled H_2_O 1 l), YM agar, and synthetic nutrient agar (SNA, sucrose 0.2 g, dextrose 0.2 g, KH_2_PO_4_ 1 g, KNO_3_1 g, MgSO_4_.7H_2_O 0.5 g, KCl 0.5 g, agar 20 g, distilled H_2_O 1 l). Conidiophores and conidia (Fig. 5, 6) were compared and photographed from cultures grown on SNA.

### Conditions for Mass Spectrometry and Analysis of Nodulisporic Acids

To prepare the samples for liquid chromatography mass spectrometry (LC-MS) analysis, fermentations were extracted with equal volumes of acetone, and the extracts were filtered and concentrated by evaporation under N_2_ to about 2× concentration relative to the original fermentation prior LC-MS analysis. In a first line identification, 2 µl of the fermentation extracts were examined by LC-MS and compared to a proprietary database with signals from authentic samples of different NAs (NA A, A_1_, C_2_, D_1_, and F) obtained on an identical LC-MS system (Fig. S2). Extracts were analyzed with an Agilent (Santa Clara, CA) 1100 single quadrupole LC-MS, using a Zorbax SB-C8 column (2.1×30 mm), maintained at 40°C and with a flow rate of 300 µl min-1. Solvent A consisted of 10% acetronitrile and 90% water with 1.3 mM trifluoroacetic acid and ammonium formate, and solvent B was 90% acetronitrile and 10% water with 1.3 mM trifluoroacetic acid and ammonium formate. The gradient started at 10% B and went to 100% B in 6 min, constant at 100% B for 2 min and returned to 10% B for 2 min to initialize the system. Full diode array UV scans from 100 to 900 nm were collected in 4 nm steps at 0.25 sec/scan. The eluting solvent was ionized using the standard Agilent 1100 electrospray ionization source adjusted to a drying gas flow of 11 l min-1 at 325°C and a nebulizer pressure of 40 psig. The capillary voltage was set to 3500 V. Mass spectra were collected as full scans from 150 *m/z* to 1500 *m/z*, with one scan every 0.77 sec, in both positive and negative modes. The DAD spectra, retention time, positive and negative mass spectra of the active samples were searched with an in-house developed application and compared to the UV-LC-MS data from known metabolites stored in a proprietary database [58], [62] where metabolite standard data [3], [12], [13], [14] had been obtained using the same LC-MS conditions.

A subset of extract samples identified as containing NAs (1 µl) were further vacumn concentrated to about 5×. The presence and relative abundance of different metabolites were verified in these fermentation extracts by HPLC-high resolution-electrospray ionization-mass spectrometry recorded on a Bruker maXisTM QTOF-MS instrument (Bruker Daltronics GmbH, Bremen, Germany) coupled to the same HPLC system as described above.

### DNA Extraction, PCR, Sequencing, Phylogenetic Analysis

Genomic nucleic acids were extracted as previously described [20], [36]. The ITS1-5.8S-ITS2 region was amplified using universal primers ITS4 and ITS5 [63]. The β-tubulin and a fragment of the α-actin genes were obtained respectively using PCR primers T1/T22 [64] and ACT-512F/ACT-783R [65]. PCR reactions were carried out following standard procedures (40 cycles of 30 s at 93°C, 30 s at 53°C and 2 min at 72°C). About 0.1 µg/ml of the double stranded amplification products were sequenced using the ABI PRISM Dye Terminator Cycle Sequencing Ready Reaction Kit (Perkin Elmer, Norwalk CT) following the procedures recommended by the manufacturer. Purified PCR products were directly sequenced using the same primer pairs as in the PCR reactions. Inner primers (T21, T10, T11, T12, T224) were used to complete the β-tubulin gene sequence [64]. An additional primer bt_1 5′-CTTATGTTTACTGCTGACCC-3′ derived from the 5′ end of an initial alignment of the β-tubulin sequences was designed in our laboratory to amplify the DNA of the species that failed in the initial attempt. Partial sequences obtained in sequencing reactions were assembled with Genestudio 2.1.1.5. (Genestudio, Inc., Suwanee, GA, USA). Individual and concatenated DNA sequences were aligned with Genestudio 2.1.1.5 and the alignments were deposited in TreeBASE (SN 12827). Phylogenetic relationships were reconstructed from separate alignments including each the three genes. Furthermore, an alignment of concatenated ITS, α-actin, and β-tublin sequences was analyzed that included those strains for which all three genes had been sequenced.

Bayesian inference and Markov chain Monte Carlo simulations (MCMC) were used for estimating phylogenetic hypotheses from individual and combined gene data sets, and were implemented in the program MrBayes 3.01 (http://mrbayes.sourceforge.net) [66]. To improve chain mixing, four incrementally heated simultaneous MCMCs were run over 2,000,000 generations. MrModeltest 2.2 [67] was used to perform hierarchical likelihood ratio tests to calculate the Akaike information criterion (AIC) values of the nucleotide substitution models. The model selected by AIC and for the alignment of the separate β-tubulin and α-actin gene fragments was the model allowing two classes of substitution types, a portion of invariant alignment positions, and mean substitution rates varying across the remaining positions according to a gamma distribution. Analysis of the DNA sequence alignment resulting from concatenation of the three genes was based on the HKY+I+G model. Priors used for the MCMC processes were a Dirichlet distribution for substitution rates and nucleotide frequencies, and a uniform prior for the rate parameter of the gamma distribution. For all phylogenetic analyses, the sampling frequency at which the trees were stored was 100, the 1000 first trees were discarded, and a majority rule consensus tree was calculated.

Because NAs-producing fungi were widespread, we predicted that other investigators, working with tropical endophytes would have encountered the fungus and deposited ITS sequences in GenBank. GenBank was interrogated with sequence homology searches using the different variations of their unedited ITS sequences. These sequences were then aligned, as described above, with the core *H. pulicicidum* ITS sequences and a tree was calculated.

In addition, bootstrap support values for all trees were assessed calculating 500 replicates with RAxML v7.0.3 [68]. All parameters were estimated by the software.

### Nomenclature

The electronic version of this article in Portable Document Format (PDF) in a work with an ISSN or ISBN will represent a published work according to the International Code of Nomenclature for algae, fungi, and plants, and hence the new names contained in the electronic publication of a PLOS ONE article are effectively published under that Code from the electronic edition alone, so there is no longer any need to provide printed copies.

In addition, the new name contained in this work has been submitted to MycoBank from where it will be made available to the Global Names Index. The unique MycoBank number can be resolved, and the associated information viewed through any standard web browser by appending the MycoBank number contained in this publication to the prefix http://www.mycobank.org/MB/. The online version of this work is archived and available from the following digital repositories: PubMed Central, LOCKSS.

## Supporting Information

Figure S1 **A.** Phylogenetic tree of *Hypoxylon pulicicidum* and related species of the Xylariaceae inferred from Bayesian analysis of ß-tubulin partial sequences. *Biscogniauxia anceps* was designated the outgroup. Clade probability values/maximum likelihood bootstrap values are indicated respectively at the branches. Values <50 are designated by -. Bar represents 10 changes. B. Phylogenetic tree of *H. pulicicidum* and related species of the Xylariaceae inferred from Bayesian analysis of α-actin partial sequences. *B. anceps* was designated the outgroup. Clade probability values/maximum likelihood bootstrap values are indicated respectively at the branches. Values <50 are designated by -. Bar represents 10 changes.
(PDF)

Figure S2 **Identification of nodulisporic acid A in fermentation extracts of strains of** ***Hypoxylon pulicicidum*** **A1.** UV spectrum of nodulisporic acid A with retention time of elution (6.28 min). A2. Positive ion mass spectrum of nodulisporic acid A (MW = 679 Da) at the same retention time. The combination of A1, A2 and retention time produces the fingerprint of nodulisporic acid A. See methods for LC–MS protocols. B1, C1, D1, E1, F1 and G1 are the UV spectra of extracts of the six strains at the corresponding retention time, and B2, C2, D2, E2, F2 and G2 are the positive ion mass spectra of the respective sample at the indicated retention time. Positive ion mass spectra indicated presence of nodulisporic acid A in all samples. UV spectrum for MF5954 was very similar to nodulisporic acid A, but the rest of the signals were near baseline, however, all retention time were consistent with that from nodulisporic acid A.
(PDF)

Figure S3 **Incomplete conidial germination in** ***Hypoxylon pulicicidum*** **and germination of conidial and hyphal fragments.** MF5954 arrested conidial germination. MUCL 49879 complete failure of germination.
(PDF)

Figure S4 **Illustration of** ***Hypoxylon investiens*** **(MJF 10128) and a list of addition specimens examined.**
(PDF)
