# Supplementary figures and images for: Hypoxylon pulicicidum sp. nov. (Ascomycota, Xylariales), a Pantropical Insecticide-Producing Endophyte
Source: PLoS One. 2012 Oct 9;7(10):e46687. doi: 10.1371/journal.pone.0046687 (PMC3467290; doi:10.1371/journal.pone.0046687)

A

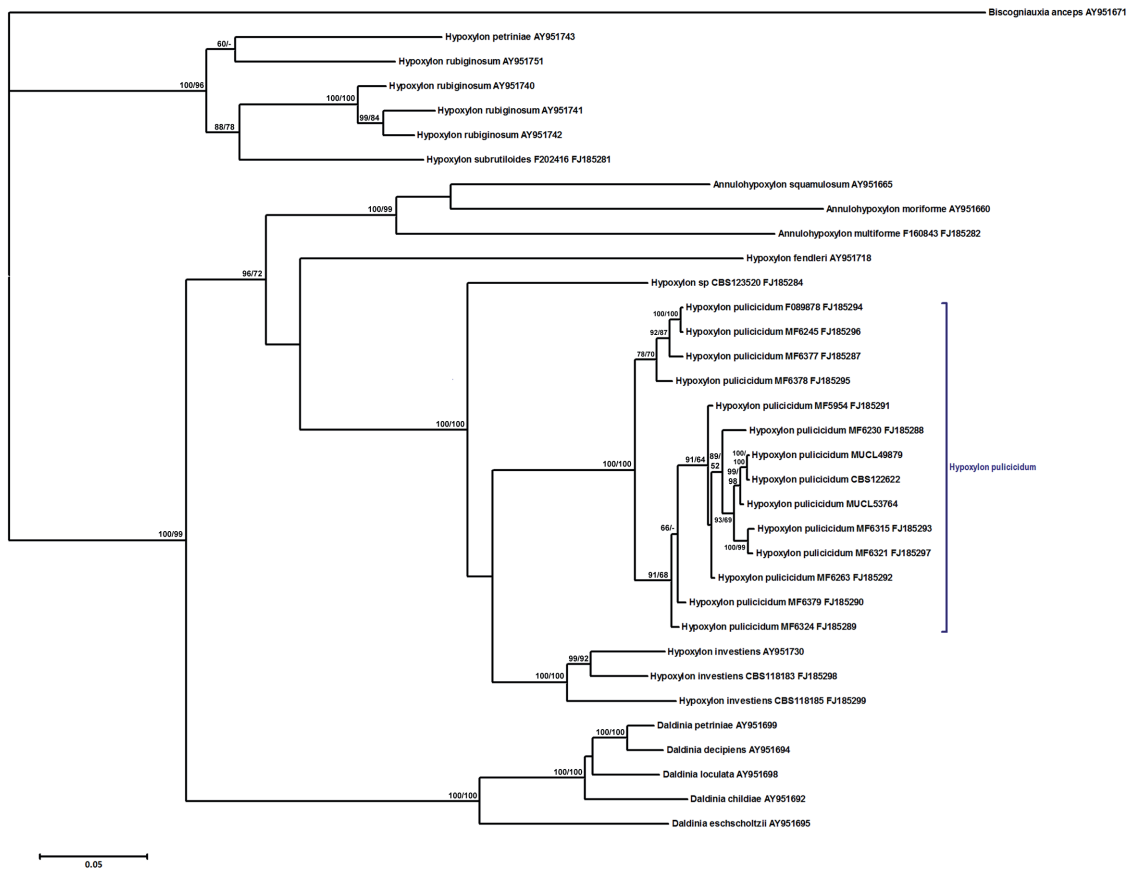

B

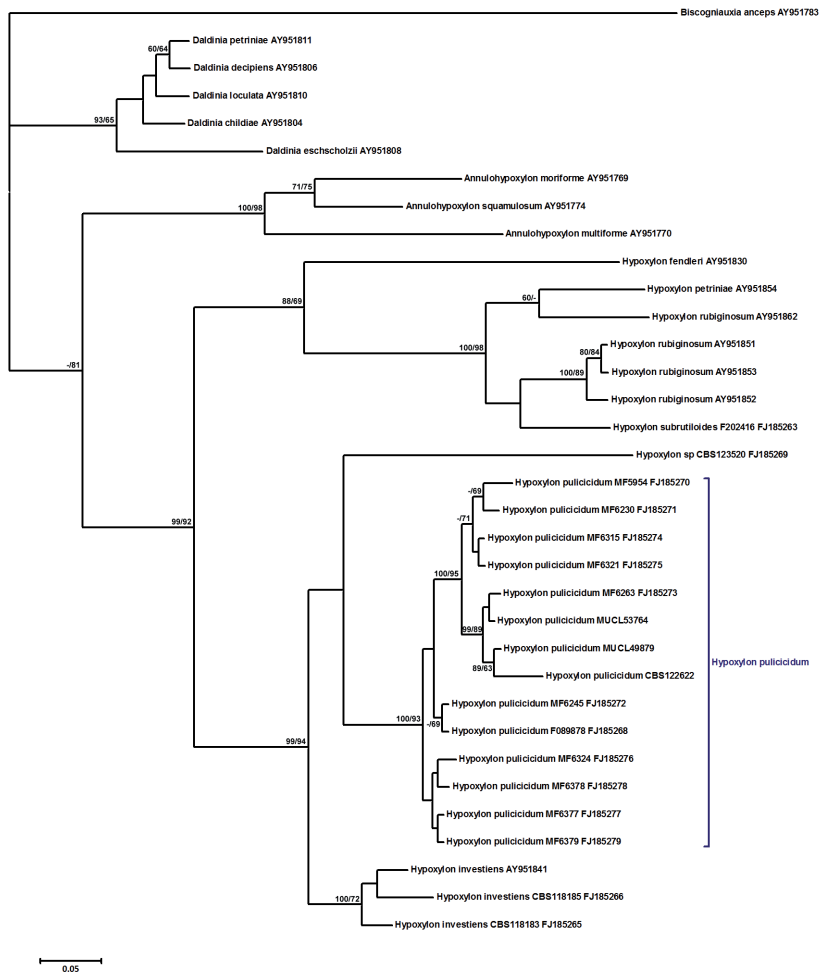

Supplement: Figure S1 — A. Phylogenetic tree of Hypoxylon pulicicidum and related species of the Xylariaceae inferred from Bayesian analysis of ß-tubulin partial sequences. Biscogniauxia anceps was designated the outgroup. Clade probability values/maximum likelihood bootstrap values are indicated respectively at the branches. Values <50 are designated by -. Bar represents 10 changes. B. Phylogenetic tree of H. pulicicidum and related species of the Xylariaceae inferred from Bayesian analysis of α-actin partial sequences. B. anceps was designated the outgroup. Clade probability values/maximum likelihood bootstrap values are indicated respectively at the branches. Values <50 are designated by -. Bar represents 10 changes. (PDF) [file pone.0046687.s001.pdf]

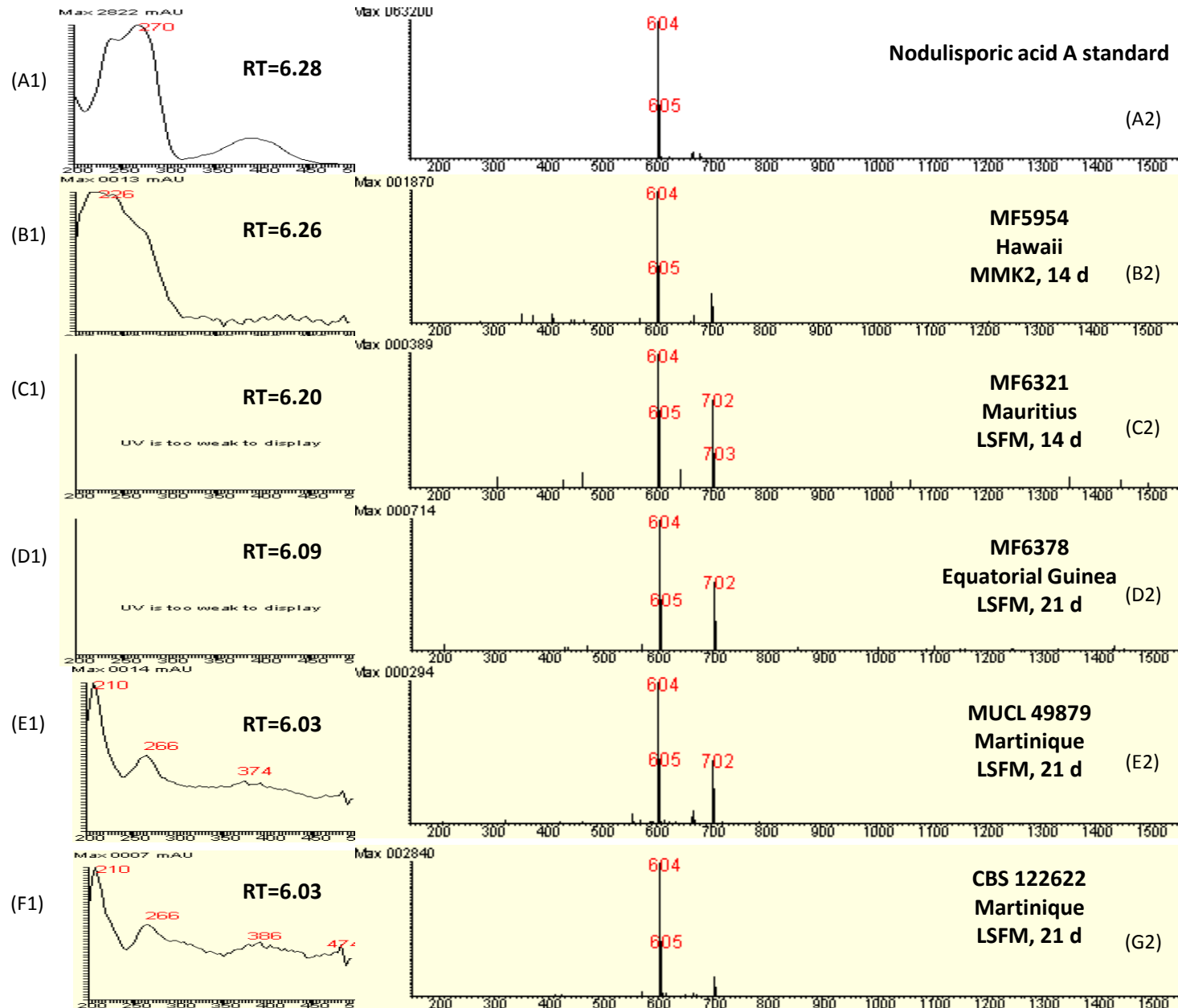

Supplement: Figure S2 — Identification of nodulisporic acid A in fermentation extracts of strains of Hypoxylon pulicicidum A1. UV spectrum of nodulisporic acid A with retention time of elution (6.28 min). A2. Positive ion mass spectrum of nodulisporic acid A (MW = 679 Da) at the same retention time. The combination of A1, A2 and retention time produces the fingerprint of nodulisporic acid A. See methods for LC–MS protocols. B1, C1, D1, E1, F1 and G1 are the UV spectra of extracts of the six strains at the corresponding retention time, and B2, C2, D2, E2, F2 and G2 are the positive ion mass spectra of the respective sample at the indicated retention time. Positive ion mass spectra indicated presence of nodulisporic acid A in all samples. UV spectrum for MF5954 was very similar to nodulisporic acid A, but the rest of the signals were near baseline, however, all retention time were consistent with that from nodulisporic acid A. (PDF) [file pone.0046687.s002.pdf]
