# Supplementary material for: Hypoxylon pulicicidum sp. nov. (Ascomycota, Xylariales), a Pantropical Insecticide-Producing Endophyte
Source: PLoS One. 2012 Oct 9;7(10):e46687. doi: 10.1371/journal.pone.0046687 (PMC3467290; doi:10.1371/journal.pone.0046687)

# *Hypoxylon pulicicidum* MUCL 49879
# Conidia after 48 hr on Cornmeal Dextrose agar

Conidia don't germinate

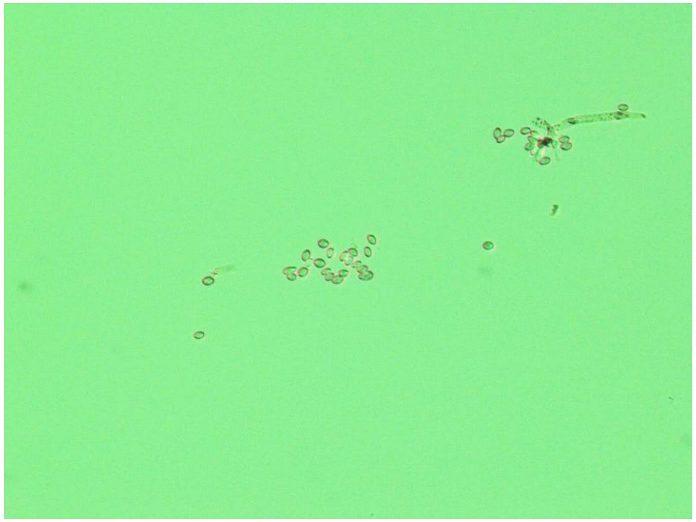

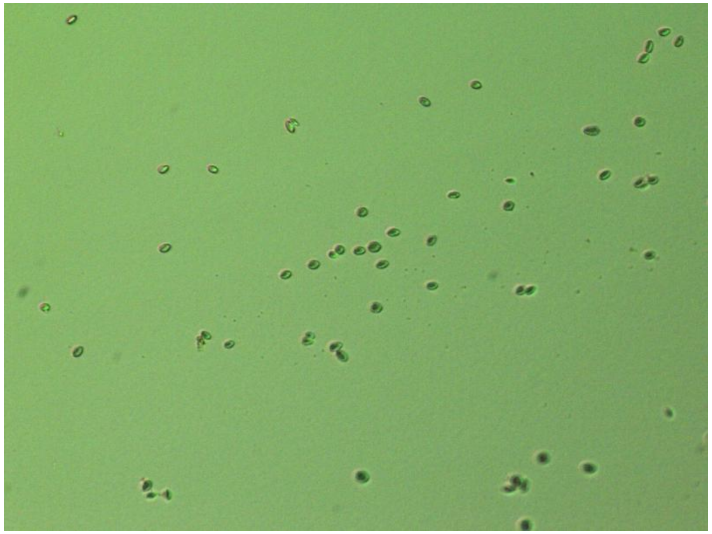

Conidiophores and hyphal fragments germinate

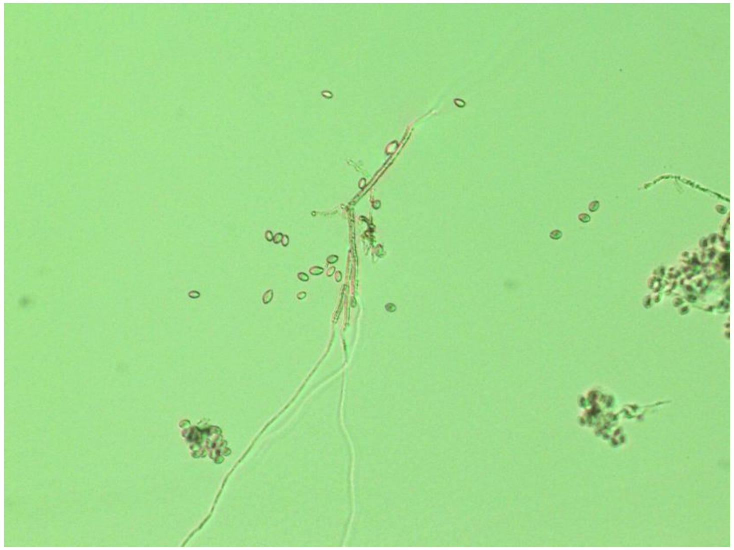

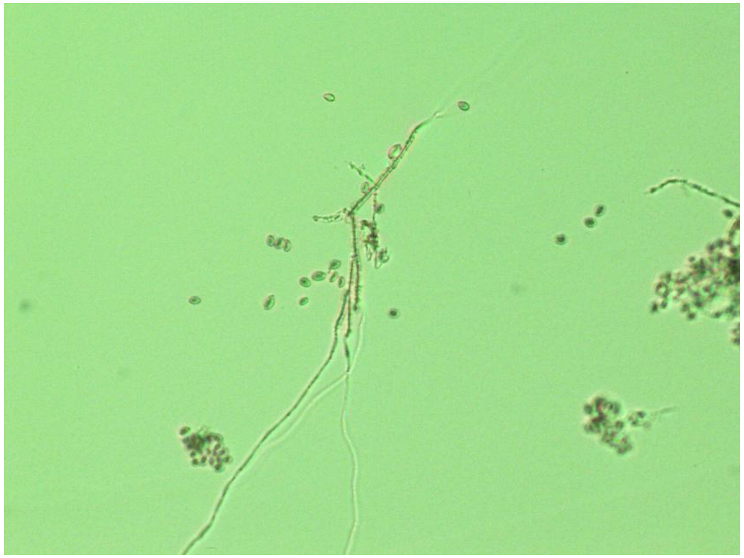

# *Hypoxylon pulicicidum* MF 5954
# Conidia after 48 hr on cornmeal dextrose agar

Conidia only form germ tube

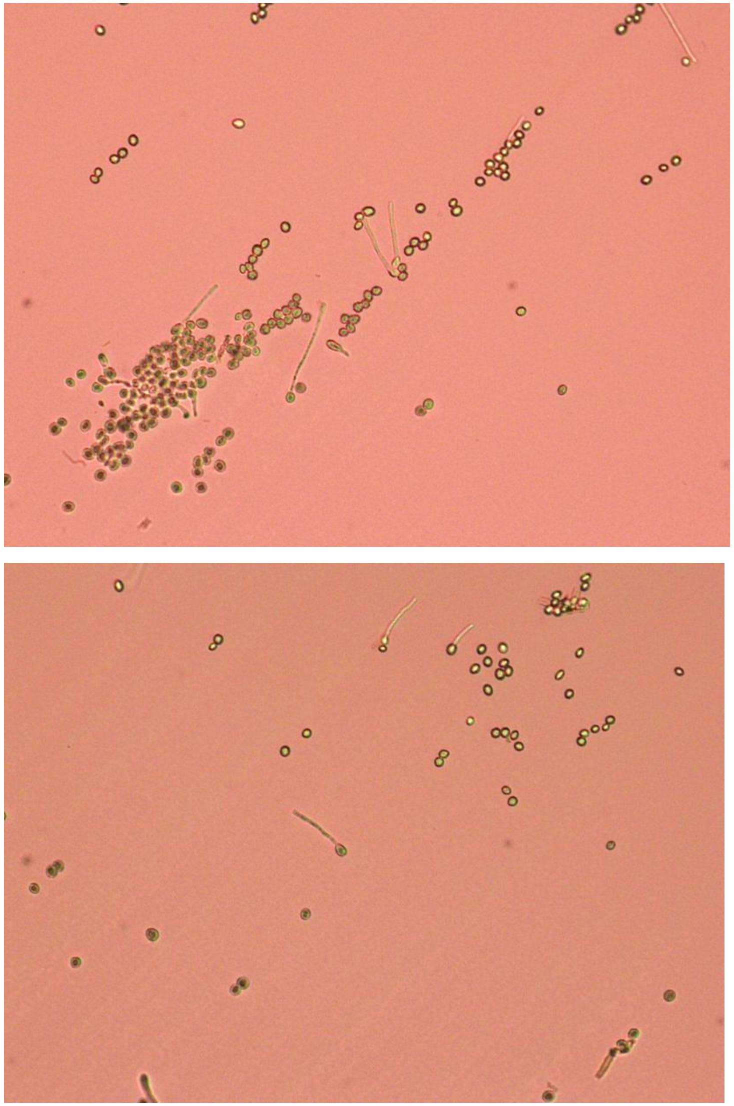

Conidiophores and hyphal fragments germinate

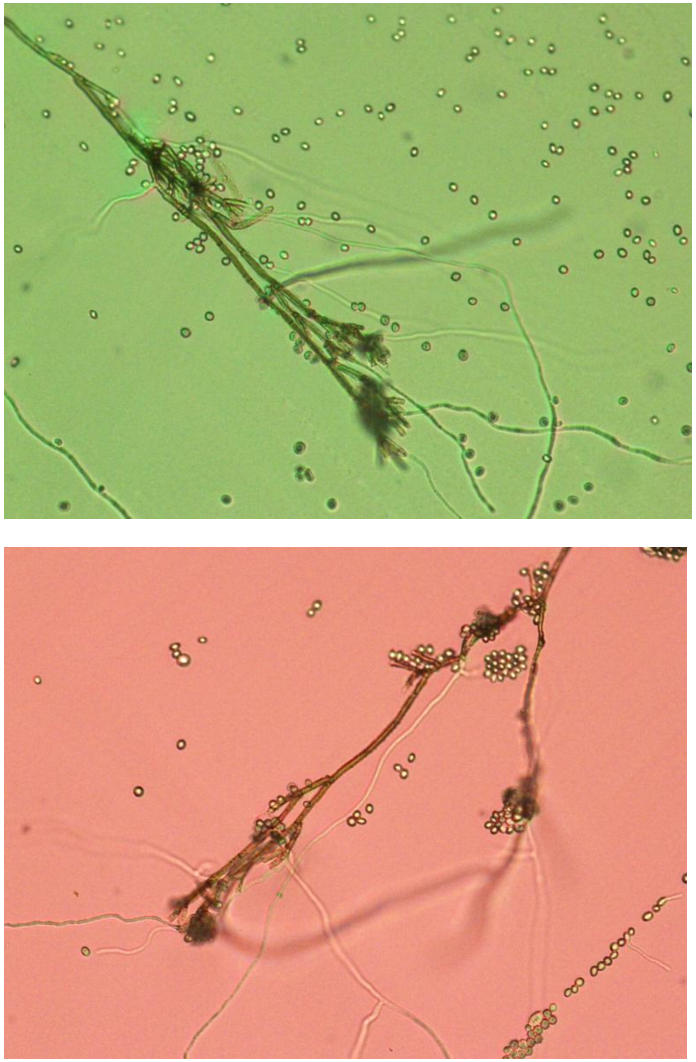

Supplement: Figure S3 — Incomplete conidial germination in Hypoxylon pulicicidum and germination of conidial and hyphal fragments. MF5954 arrested conidial germination. MUCL 49879 complete failure of germination. (PDF) [file pone.0046687.s003.pdf]
