# Supplementary material for: Hypoxylon pulicicidum sp. nov. (Ascomycota, Xylariales), a Pantropical Insecticide-Producing Endophyte
Source: PLoS One. 2012 Oct 9;7(10):e46687. doi: 10.1371/journal.pone.0046687 (PMC3467290; doi:10.1371/journal.pone.0046687)
Supplement: Figure S4 — Illustration of Hypoxylon investiens (MJF 10128) and a list of addition specimens examined. (PDF) [file pone.0046687.s004.pdf]

Supplementary Figure S3. *Hypoxylon investiens*, illustration and list of material examined. *H. investiens* MJF 10128. 1A. Close up of stromatal surface. 1B. Cross section of stroma and perthecia.1C. KOH-extractable pigments. 1D. Ascospores. 1E. Ascus apical apparatus stained in Melzer's regent.

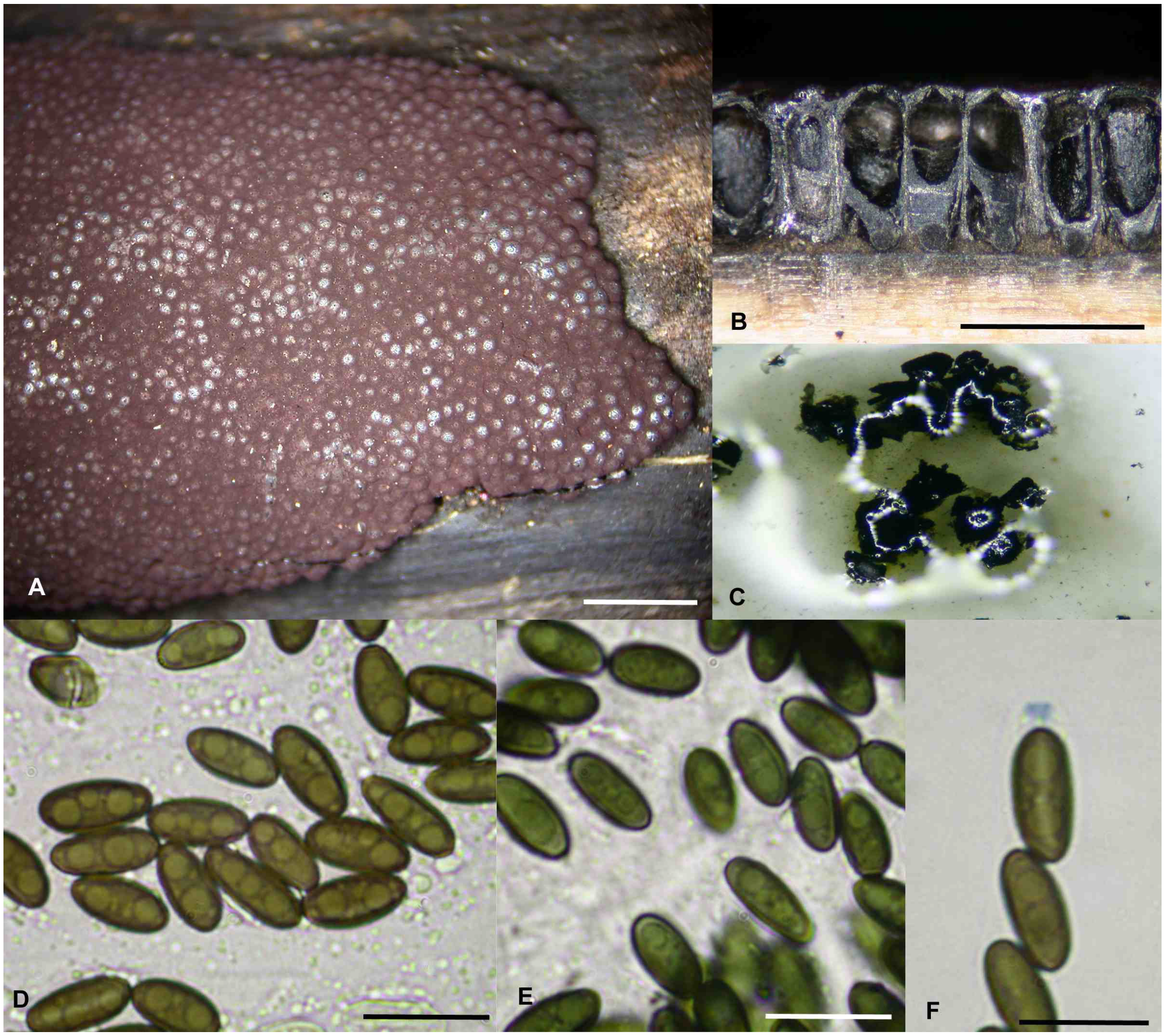

Additional Specimens Examined

Material studied for comparison of Hypoxylon investiens (Schwein.) M.A. Curtis s. str.
Cuba: La Habana Prov. Managua, Mar. 3 1905, Earle & Murrill 90 (NY, see also Ju & Rogers 1996 as *H. investiens*).
French Guiana, Saül, on the trail to Mt. Calbao, 200-300 m elev., Feb. 1986, G. J. Samuels, G. J. 3710 (NY, see Ju & Rogers 1996 as *H. investiens*); Sinnamary, Piste Saint Elie, on a corticated branch 22 Apr. 2010, leg Christian Lechat, CLL10011 (LIP, culture MUCL53758).
French West Indian Islands, Martinique: Le Robert, Pointe Bateau, coastal xerophilic to mesophilic forest, dead blackened wood, 26 Aug. 2010, J. Fournier MJF 10128 (LIP). Prêcheur, Anse Couleuvre, blackened wood of dictyledons, 24 Aug. 2010, J. Fournier MJF 10075 (LIP); Prêcheur, Anse Couleuvre/ 24 Aug. 2010, blackened wood of *Cecropia*

schreberiana, J. Fournier MJF10083 (LIP, culture MUCL 53307); Saint Esprit, Bois La Charles, bark of dicotyledons, 30 Aug. 2010, J. Fournier MJF10263 (LIP, culture MUCL 53316).

Malaysia, Sabah, exact locality unknown, 2003, T. Laessoe TL-6003 (C, culture CBS 118183; see Platas et al. 2008 and Bitzer et al. 2008; GenBank DNA sequences FJ185265, FJ185298 and FJ185307).

Mexico, San Blas, Dec. 1962, P. Martin 1547 (NY); Dec. 1961, P. Martin 953 and 959 (see Martin 1969 as *H. investiens*).

Puerto Rico, Rio Piedras, Dec 4, 1915, J. A. Stevenson as *H. rubiginosum* (NY).

Taiwan, Taipei Co., Wu-lai, Hsin-hsien, 29.06.2000, on wood, Y-M. Ju, YMJ 89062905 (HAST, see Hellwig et al. 2005). Note - used for HPLC comparisons in Figure 9.

USA, Louisiana, Martinsville, 30 July 1899, J. B. Langlois, Flora Ludoviciana 2112, as *H. rubiginosum* (NY); Missouri, Lake Ozark Co., Dec. 1961, P Martin 1169, see Martin (1969) as *H. murcidum* (NY); Missouri, Boone Co., Columbia, Aug. 1962, P Martin 1097 see Martin (1969) as *H. murcidum* (NY); North Carolina, Salem & Pennsylvania, Bethlehem, decorticated wood, Syn. 1210, (PH, **holotype** of *Sphaeria investiens*); same coll. data, Collins Coll. 35, decorticated wood (PH, **isotype** of *Sphaeria investiens*); North Carolina, Salem, 1921, C. L. Shear as *H. rubiginosum* (NY).
